# Association of size for gestational age and dehydroepiandrosterone sulfate with cardiometabolic risk in central precocious puberty girls

**DOI:** 10.3389/fendo.2023.1131438

**Published:** 2023-05-24

**Authors:** Guijiao Zhang, Huan Yu, Shengxu Yu, Xiaoping Luo, Yan Liang, Ling Hou, Wei Wu

**Affiliations:** Department of Pediatrics, Tongji Hospital, Tongji Medical College, Huazhong University of Science and Technology, Wuhan, China

**Keywords:** children, central precocious puberty, birth weight for gestational age, dehydroepiandrosterone sulfate, cardiometabolic risk

## Abstract

**Objective:**

The aim of this study was to assess whether size for gestational age and dehydroepiandrosterone sulfate (DHEAS) are associated with cardiometabolic risk in central precocious puberty (CPP) girls.

**Methods:**

The retrospective study included 443 patients with newly diagnosed CPP. Subjects were categorized by birth weight for gestational age (appropriate [AGA], small [SGA], and large [LGA] for gestational age) and serum DHEAS concentration (high [≥75th percentile] and normal [<75th percentile] DHEAS). Cardiometabolic parameters were examined. Composite cardiometabolic risk (CMR) score was calculated based on BMI, blood pressure, glucose, insulin, triglyceride, and HDL cholesterol. Non-obesity CMR score was computed, omitting the value from BMI. Logistic regression models, general linear models, and partial correlation analyses were used to evaluate associations. Propensity score matching was performed for sensitivity analyses.

**Results:**

Overall, 309 patients (69.8%) were born AGA, 80 (18.1%) were born SGA, and 54 (12.2%) were born LGA. Compared with AGA counterparts, CPP girls born SGA were more prone to have elevated HbA1c (adjusted OR = 4.54; 95% CI, 1.43–14.42) and low HDL cholesterol (adjusted OR = 2.33; 95% CI, 1.18–4.61). In contrast, being born LGA was not associated with increased risk for any glucose or lipid derangements. Despite the fact that elevated CMR score was more common among individuals born LGA than AGA (adjusted OR = 1.84; 95% CI, 1.07–4.35), no significant difference was found on non-obesity CMR score (adjusted OR = 0.75; 95% CI, 0.30–1.88). When controlling for age, birth weight SDS, and current BMI-SDS, individuals with high DHEAS exhibited higher HDL cholesterol and apolipoprotein A-1 concentrations and lower triglyceride level and non-obesity CMR score. Furthermore, DHEAS correlated positively with HDL cholesterol and apolipoprotein A-1 and negatively with triglyceride, prominently in girls born SGA, after adjustments for the three abovementioned confounders. Sensitivity analyses corroborated the findings.

**Conclusion:**

Among CPP girls, those born SGA were more likely to possess cardiometabolic risk factors compared to their AGA peers. The difference we observed in cardiometabolic risk between individuals born LGA and AGA was driven by BMI. High DHEAS was associated with favorable lipid profile in CPP girls, even in subjects born SGA.

## 1 Introduction

Growing evidence indicates that puberty onset has been occurring at an earlier age (1). In parallel with this trend, an increased number of children (mainly girls) has been referred for evaluation for suspected precocious puberty (2). It has been reported that individuals with early timing of puberty have an increased risk for adult metabolic syndrome-related derangements (3). One recent study provided preliminary evidence supporting altered metabolic pathway in central precocious puberty (CPP) girls (4). However, not all children with CPP develop associated comorbidities. Identifying those patients at high risk and intervening in time is a clinical priority in childhood.

Environmental changes taking place in critical periods of development could determine many physiological adaptations. Size for gestational age, an acknowledged proxy measure of intrauterine growth, has an impact on later health, through altered growth trajectories and weight status (5). Previous research reported that compared with appropriate for gestational age (AGA) counterparts, girls born small for gestational age (SGA) reached all pubertal markers at an earlier age and likewise being born large for gestational age (LGA) experienced an earlier pubertal take-off, although somewhat controversial (6, 7). To which extent birth size contributes to the risk for cardiometabolic disorders in girls with CPP remains unclear.

Recent studies revealed an early origin of adrenarche and demonstrated that prepubertal girls with high dehydroepiandrosterone sulfate (DHEAS) concentration was associated with early pubertal maturation (8–11). One study found a positive association between premature adrenarche (PA) and increased cardiometabolic risk in SGA girls (12). In contrast, emerging evidence suggested that high DHEAS level served as a protective role for several cardiometabolic risk factors in prepubertal children with normal birth weight (13, 14). An attractive and yet unresolved question is the link between DHEAS and metabolic health and whether this association was modified by birth size.

The current study aimed to investigate the association of SGA or LGA with cardiometabolic risk in CPP girls and evaluate the relationship between circulating DHEAS and cardiometabolic parameters among patients stratified for birth weight status.

## 2 Materials and methods

### 2.1 Study population

The retrospective study was conducted by reviewing the medical records of 803 girls (age ≤9 years) who were admitted to our center for suspected precocious puberty between October 2017 and October 2020. Patients were comprehensively assessed based on clinical, imaging, and laboratory criteria issued by the Chinese Medical Association in 2015 (15). CPP was diagnosed if girls developed secondary sexual characteristics before the age of 8 years, accompanied by growth spurt and advanced bone maturation (15). Gonadal axis function initiation was presented by a positive gonadotropin-releasing hormone (GnRH) provocation test [peak luteinizing hormone (LH) ≥5 IU/L and peak LH/peak follicle-stimulating hormone (FSH) ≥0.6] or basal levels of LH ≥5 IU/L (15). The pelvic ultrasound examination showed uterine length exceeding 3.4 cm, ovarian volume >1 ml, and multiple follicles ≥4 mm in diameter (15). Children were excluded if they had peripheral precocious puberty, normal variant puberty (premature thelarche and PA), rapidly progressive puberty, secondary to central nervous system abnormalities (e.g., space-occupying lesion, acquired abnormalities, and congenital developmental malformations), other types of abnormal growth and development (e.g., abnormal thyroid function and pre-existing renal disease and adrenal disease), previous or current use of other medication, and no gestation age or birth weight records (**Supplementary Figure S1**). A total of 443 patients were finally included.

The study was approved by the Ethics Committee of Tongji Hospital of Tongji Medical College of Huazhong University of Science and Technology (TJ-IRB20211011).

### 2.2 Grouping

Gestational age and birth weight were based on parent recall. Size for gestational age was derived by applying the reference birth weight curves for the Chinese population released on 2020 (16). Subjects were analyzed categorically using the following three groups: SGA (below the 10th percentile), AGA (between the 10th and 90th percentile), and LGA (above the 90th percentile). The group with high DHEAS concentration was defined as individuals with value above the 4th quartile (≥1,290 nmol/L, ≥48 μg/dl, higher than the cutoff for biochemical adrenarche) and the group with normal DHEAS concentration as those with value below the 4th quartile based on overall sample.

### 2.3 Outcome measures

Puberty, a sensitive period characterized by hormonal changes, plays an essential role in metabolism and body composition changes. Previous studies indicated that girls with CPP at diagnosis had a higher risk of developing cardiometabolic disorders (17, 18). Thus, it may be beneficial to assess metabolic profiles at diagnosis. In our study, composite cardiometabolic risk (CMR) score was calculated as the total study population (AGA, SGA, and LGA) age- and sex-standardized *z* score of the following components by summing body mass index (BMI), the mean of systolic blood pressure (SBP), and diastolic blood pressure (DBP), ln triglyceride, fasting glucose, square root of fasting insulin, and high-density lipoprotein (HDL) cholesterol (multiplied by −1), then dividing this value by 6 (19, 20). Non-obesity CMR score was computed excluding the *z* score of BMI. Higher CMR score meant increased cardiometabolic event risk. Elevated CMR score was defined in individuals with a value above the highest quartile and elevated non-obesity CMR score was defined similarly. The CMR score and non-obesity CMR score were only calculated for children with data available for all components. Triglyceride and insulin were not normally distributed and thus were transformed before standardization.

Overweight was defined as ≥85th percentile and obesity as ≥95th percentile of age- and sex-specific BMI based on the growth charts of children in China (21). High blood pressure was defined as SBP or DBP ≥95th percentile for age, sex, and height (22). Hyperglycemia was defined as high fasting glucose (≥5.6 mmol/L) and elevated hemoglobin A1c (HbA1c) (>5.7%) (23). Homeostasis model of assessment for insulin resistance (HOMA-IR) was calculated as the product of fasting glucose (mmol/L) and fasting insulin (IU/L) divided by 22.5 (24). A value of HOMA-IR >3.00 was used as cutoff of IR (25). According to the recommendation from NHLBI (National Heart, Lung, and Blood Institute), high total cholesterol (TC) was defined as ≥5.18 mmol/L; high low-density lipoprotein (LDL) cholesterol as ≥3.37 mmol/L; low HDL cholesterol as <1.04 mmol/L; high non-HDL cholesterol as ≥3.76 mmol/L; high triglyceride as ≥1.13 mmol/L; high apolipoprotein B as ≥1.10 g/L; low apolipoprotein A-1 as <1.15 g/L; and high lipoprotein (a) as ≥72 nmol/L (22).

### 2.4 Confounding variables

Confounding variables were selected based on previous literature and gathered from routinely documented medical information (14, 26, 27). Preterm birth, feeding type (exclusive breastfeeding, formula feeding, or mixed feeding), disease during pregnancy (gestational diabetes mellitus or gestational hypertension), and family history of cardiometabolic diseases (parents or grandparents with diabetes, hypertension, or hyperlipidemia) were examined as confounders when investigating the link between birth size and cardiometabolic risk. We also adjusted for child’s age and puberty stage when the outcome was measured. When exploring the association of DHEAS and cardiometabolic profiles, age, BMI-SDS, and SDS for birth weight (BW-SDS) were adjusted.

### 2.5 Other variables’ definition

Anthropometric measures were carried out by trained professionals. Patients were evaluated in barefoot with light clothing. Standing height was measured to the nearest 0.1 cm using a stature meter. Weight was measured to the nearest 0.1 kg using a digital scale. Secondary sexual characteristic examination was performed by trained pediatric endocrinologists. Breast development was evaluated by inspection and palpation, and pubic hair development was evaluated by visual examination and then assigned according to Tanner stage. The genetic target height (THt) was calculated as the average height of both parents minus 6.5 cm. The predicted adult height (PAH) was estimated based on the Bayley–Pinneau method (28). SDS for height, THt, and PAH were calculated to correct for age and sex based on Chinese growth reference data (29). The difference between PAH-SDS and THt-SDS (PAH-SDS_THt-SDS_) was used to predicted the loss in height potential.

### 2.6 Biochemical analysis

Overnight fasting venous blood samples were obtained from 8:00 a.m. to 10:00 a.m. after resting for 30 min and tested as previously described (30). Generally, fasting glucose was measured using the hexokinase method, fasting insulin was determined using chemiluminescence detection, and HbA1c was measured using high-performance liquid chromatography. The lipoprotein cholesterol and apolipoprotein levels were detected by direct enzymatic and immunoturbidimetric methods, respectively. GnRH stimulation test was performed and sex steroid hormone concentrations (LH, FSH, estradiol, DHEAS, and androstenedione) were all measured by chemiluminescence enzyme immunoassay.

### 2.7 Statistical analysis

Statistical analyses were conducted using SPSS software (version 22.0.; IBM Corp., Armonk, NY) and SAS (version 9.1; SAS, Inc., Cary, NC). Graphs were generated using GraphPad Prism (version 8; GraphPad, San Diego, CA). Normality and linearity of each variable were assessed, and variables were transformed when necessary. The optimal transformation was determined by the skewness and kurtosis. Categorical and continuous variables were presented as frequency (percentage), mean ± standard deviation and median (interquartile range), as appropriate unless indicated. Clinical characteristics and sex hormone profiles stratified for birth weight status were compared by one-way analysis of variance (Sidak correction) or Kruskal–Wallis nonparametric test and chi-square test or Fisher exact test as appropriate. Association between birth size and adverse levels of cardiometabolic risk factors was examined using unadjusted and adjusted logistic regression. Differences in cardiometabolic parameters between the high and normal DHEAS groups were investigated by independent samples *t*-test and analysis of covariance. The correlation between DHEAS and cardiometabolic risk factors was analyzed by partial correlation analyses. The specific test was stated in the legends. Statistical significance was based on *P* value less than 0.05.

Sensitivity analyses were conducted using a stricter definition of SGA (<−2SDS) and LGA (>2SDS), and ruling out participants with preterm or having missing data. The extreme birth size (SGA or LGA) as cases and AGA individuals as control were matched on their propensity score with nearest-neighbor matching at a 1:3 ratio without replacement, respectively. Matching factors included child’s age, BMI-SDS, puberty stage, feeding pattern, disease during pregnancy, and family history of cardiometabolic diseases.

## 3 Results

### 3.1 Subject characteristics

Characteristics of the sample stratified by birth weight for gestational age are shown in **Table 1**. Overall, 309 patients (69.8%) were born AGA, 80 (18.1%) were born SGA, and 54 (12.2%) were born LGA. A total of 27 participants were born preterm and the occurrence of prematurity was similar among the AGA, SGA, and LGA groups (17 [5.5%] vs. 7 [8.8%] vs. 3 [5.6%], *p* = 0.55). Type of feeding appeared to differ somewhat across three categories (*p* = 0.08). Further analysis confirmed that the exclusive breastfeeding rate was much lower in SGA (*p*_SGA vs. AGA_ = 0.006, *p*_SGA vs. LGA_ = 0.02). The proportions of complications during pregnancy, primiparous mothers, and mode of delivery had equal distribution among groups. There was no suggestion that family history of early menarche and cardiometabolic diseases differed among the three groups.

**Table 1:** General characteristics stratified for size for gestational age in CPP girls.

Characteristics	Overall (*n* = 443)	AGA (*n* = 309)	SGA (*n* = 80)	LGA (*n* = 54)	*p-*Value
Child					
Age, years	7.9 (7.3, 8.4)	7.9 (7.4, 8.4)	7.8 (7.3, 8.6)	7.9 (7.0, 8.4)	0.86^†^
Height-for-age, cm	130.6 ± 7.4	131.1 ± 6.7	128.1 ± 7.4	131.8 ± 9.8	0.003^‡^
Height-for-age SDS	0.65 ± 1.04	0.69 ± 1.00	0.20 ± 1.03	1.12 ± 1.08	<0.001^‡^
THt-SDS	−0.40 ± 0.73	−0.40 ± 0.73	−0.53 ± 0.76	−0.18 ± 0.65	0.03^‡^
PAH-SDS _THt-SDS_	−0.97 ± 1.06	−0.91 ± 1.00	−1.26 ± 1.26	−0.85 ± 1.03	0.02^‡^
Weight, kg	28.0 (24.5, 32.5)	28.1 (24.6, 33.0)	26.1 (23.5, 30.0)	29.8 (26.0, 36.0)	0.002^†^
BMI, kg/m^2^	16.6 (15.3, 18.4)	16.6 (15.3, 18.3)	16.0 (14.6, 18.1)	16.9 (15.8, 19.2)	0.01^†^
BMI-SDS	0.81 ± 1.25	0.79 ± 1.22	0.59 ± 1.40	1.28 ± 1.04	0.01^‡^
BMI status					0.45^§^
Not overweight or obesity	263 (59.4%)	186 (60.2%)	51 (63.7%)	26 (48.1%)	
Overweight	66 (14.9%)	46 (14.9%)	10 (12.5%)	10 (18.5%)	
Obesity	114 (25.7%)	77 (24.9%)	19 (23.8%)	18 (33.3%)	
Breast Tanner stage					<0.001^¶^
2	285 (64.3%)	194 (62.8%)	59 (73.8%)	32 (59.3%)	
3	138 (31.2%)	108 (35.0%)	15 (18.8%)	15 (27.8%)	
4	20 (4.5%)	7 (2.3%)	6 (7.5%)	7 (13.0%)	
Pubarche	28 (6.3%)	13 (4.2%)	11 (13.8%)	4 (7.4%)	0.01^¶^
Menarche	12 (2.7%)	7 (2.3%)	2 (2.5%)	3 (5.6%)	0.33^¶^
Perinatal					
Preterm birth (<37 weeks)	27 (6.1%)	17 (5.5%)	7 (8.8%)	3 (5.6%)	0.55^¶^
Gestational age, weeks	39.0 (38.0, 40.0)	39.0 (38.0, 40.0)	39.6 (38.0, 40.0)	39.0 (38.0, 40.0)	0.57^†^
Birth weight, kg	3.2 (2.8, 3.4)	3.2 (3.0, 3.4)	2.6 (2.3, 2.7)	3.8 (3.6, 3.9)	<0.001^†^
Birth weight SDS	−0.24 (−0.91, 0.50)	−0.12 (−0.52, 0.28)	−1.72 (−1.98, −1.35)	1.36 (1.33, 1.73)	<0.001^†^
GDM or GH^a^	5 (1.2%)	2 (0.6%)	1 (1.4%)	2 (3.8%)	0.10^¶^
Primiparous	376 (84.9%)	264 (85.4%)	70 (87.5%)	42 (77.8%)	0.28^§^
Caesarean delivery	292 (65.9%)	205 (66.3%)	50 (62.5%)	37 (68.5%)	0.76^§^
Infant feeding^a^					0.08^§^
Exclusive breastfeeding	275 (64.1%)	199 (66.8%)	39 (50.0%)	37 (69.8%)	
Formula feeding	82 (19.1%)	53 (17.8%)	20 (25.6%)	9 (17.0%)	
Mixed feeding	72 (16.8%)	46 (15.4%)	19 (24.4%)	7 (13.2%)	
Family history					
Mother’s age of menarche^a^					0.92^¶^
<12 years	50 (22.3%)	39 (22.2%)	7 (28.0%)	4 (17.4%)	
12–14 years	142 (63.4%)	112 (63.6%)	15 (60.0%)	15 (65.2%)	
≥14 years	32 (14.3%)	25 (14.2%)	3 (12.0%)	4 (17.4%)	
Cardiometabolic risk^a^					0.13^¶^
Parents	15 (3.4%)	11 (3.6%)	0 (0.0%)	4 (7.7%)	
Grandparents	71 (16.2%)	54 (17.5%)	10 (13.0%)	7 (13.5%)	
No	351 (80.3%)	243 (78.9%)	67 (87.0%)	41 (78.8%)	

Continuous variables were expressed as mean ± SD or median (IQR). Categorical variables were shown as frequency (percentage). Percentages may not total 100% because of rounding. Statistical significance was based on p-value less than 0.05.

CPP, central precocious puberty; AGA, appropriate for gestational age; SGA, small for gestational age; LGA, large for gestational age; SDS, standard deviation score; THt, genetic target height; PAH, predictive adult height; PAH-SDS _THt-SDS_, the loss in height potential; BMI, body mass index; GDM, gestational diabetes mellitus; GH, gestational hypertension.

^†^Kruskal–Wallis nonparametric test;

^‡^ANOVA (Sidak correction);

^§^Chi-square test; ^¶^Fisher exact test.

a Numbers do not add up to the total value because of missing data.

Median age at first presentation was similar across groups. CPP girls born SGA exhibited lower body weight (*p* = 0.008), shorter height (*p* = 0.001), and height-for-age-SDS (*p* < 0.001), and more severe loss in predicted height potential (*p* = 0.01) when compared with subjects born AGA. THt-SDS did not vary significantly between the two groups (*p* = 0.17). Individuals born SGA were more likely to have had pubarche (*p* = 0.004) than AGA at first visit. Moreover, SGA and LGA girls showed more advanced breast development than their AGA peers, especially for Tanner stage 4 (*p*_SGA vs. AGA_ = 0.049, *p*_LGA vs. AGA_ = 0.001). For LGA subjects, the average height-for-age SDS and BMI-SDS were higher than AGA (*p* = 0.004, *p* = 0.006). Notably, there was no significant difference in the distribution of overweight/obese individuals among the three groups (*p* = 0.45).

### 3.2 Relationship between size for gestational age and cardiometabolic risk

Unadjusted and adjusted analyses for the association between birth weight for gestational age and cardiometabolic risk factors are shown in **Table 2**. Compared to individuals born AGA, being born SGA was more prone to have elevated HbA1c (adjusted OR = 4.54; 95% CI, 1.43 to 14.42) and low HDL cholesterol (adjusted OR = 2.33; 95% CI, 1.18 to 4.61). Remarkably, based on the adjusted analysis, elevated triglyceride was less common among CPP girls born SGA than those born AGA (adjusted OR = 0.31; 95% CI, 0.10 to 0.94). No association between LGA and any individual cardiometabolic risk factors was observed. Despite the fact that patients born LGA were associated with higher odds of elevated CMR score (adjusted OR = 1.84; 95% CI, 1.07 to 4.35) compared with their AGA peers, no obvious difference was found on non-obesity CMR score (adjusted OR = 0.75; 95% CI, 0.30 to 1.88).

**Table 2:** Association between size for gestational age and adverse levels of cardiometabolic risk factors.

Variable^a^	Events No. (%)	Crude (OR, 95% CI)	Adjusted (OR, 95% CI)^b^
BMI ≥95th			
AGA (*n* = 309)	78 (25.2%)	Reference	Reference
SGA (*n* = 80)	19 (23.8%)	0.93 (0.52, 1.65)	1.02 (0.50, 2.10)
LGA (*n* = 54)	18 (33.3%)	1.49 (0.80, 2.78)	1.45 (0.68, 3.07)
HbA1c >5.7%			
AGA (*n* = 304)	10 (3.3%)	Reference	Reference
SGA (*n* = 62)	6 (9.7%)	**3.15 (1.10, 9.02)**	**4.54 (1.43, 14.42)**
LGA (*n* = 50)	1 (2.0%)	0.60 (0.08, 4.79)	0.39 (0.03, 4.44)
HOMA-IR >3			
AGA (*n* = 309)	10 (3.2%)	Reference	Reference
SGA (*n* = 80)	3 (3.8%)	1.17 (0.31, 4.35)	2.41 (0.55, 10.60)
LGA (*n* = 54)	3 (5.6%)	1.76 (0.47, 6.61)	0.86 (0.14, 5.24)
HDL cholesterol <1.04 mmol/L			
AGA (*n* = 309)	43 (13.9%)	Reference	Reference
SGA (*n* = 80)	20 (25.0%)	**2.06 (1.13, 3.76)**	**2.33 (1.18, 4.61)**
LGA (*n* = 54)	11 (20.4%)	1.58 (0.76, 3.31)	1.49 (0.66, 3.37)
LDL cholesterol ≥3.37 mmol/L			
AGA (*n* = 309)	6 (1.9%)	Reference	Reference
SGA (*n* = 80)	2 (2.5%)	1.30 (0.26, 6.54)	0.97 (0.10, 8.99)
LGA (*n* = 54)	1 (1.9%)	0.95 (0.11, 8.07)	1.16 (0.13, 10.67)
Triglyceride ≥1.13 mmol/L			
AGA (*n* = 309)	50 (16.2%)	Reference	Reference
SGA (*n* = 80)	8 (10.0%)	0.58 (0.26, 1.27)	**0.31 (0.10, 0.94)**
LGA (*n* = 54)	7 (13.0%)	0.77 (0.33, 1.81)	0.68 (0.27, 1.75)
Apolipoprotein A-1 <1.15 g/L			
AGA (*n* = 309)	67 (21.7%)	Reference	Reference
SGA (*n* = 80)	23 (28.7%)	1.46 (0.84, 2.54)	1.51 (0.82, 2.78)
LGA (*n* = 54)	17 (31.5%)	1.66 (0.88, 3.13)	1.65 (0.83, 3.28)
Lipoprotein (a) ≥72 nmol/L			
AGA (*n* = 297)	39 (13.1%)	Reference	Reference
SGA (*n* = 53)	5 (9.4%)	0.69 (0.26, 1.84)	0.87 (0.32, 2.39)
LGA (*n* = 48)	6 (12.5%)	0.95 (0.38, 2.37)	0.73 (0.26, 2.09)
Blood pressure ≥95th			
AGA (*n* = 260)	53 (20.4%)	Reference	Reference
SGA (*n* = 59)	6 (10.2%)	0.44 (0.18, 1.08)	0.41 (0.15, 1.14)
LGA (*n* = 38)	8 (21.1%)	1.04 (0.45, 2.40)	1.13 (0.46, 2.81)
Elevated CMR score			
AGA (*n* = 260)	61 (23.5%)	Reference	Reference
SGA (*n* = 59)	12 (20.3%)	0.83 (0.42, 1.67)	0.69 (0.29, 1.66)
LGA (*n* = 38)	16 (42.1%)	**2.37 (1.17, 4.80)**	**1.84 (1.07, 4.35)**
Elevated non-obesity CMR score			
AGA (*n* = 260)	65 (25.0%)	Reference	Reference
SGA (*n* = 59)	13 (22.0%)	0.85 (0.43, 1.67)	0.84 (0.37, 1.90)
LGA (*n* = 38)	11 (28.9%)	1.22 (0.57, 2.60)	0.75 (0.30, 1.88)

Estimates were derived using logistic regression analyses. Bolded numbers indicate statistical significance (p < 0.05). Some variables were not shown here because OR cannot be calculated based on the number of events, in terms of fasting glucose ≥5.60 mmol/L, total cholesterol ≥5.18 mmol/L, non-HDL cholesterol ≥3.76 mmol/L, and apolipoprotein B ≥1.10 g/L.

AGA, appropriate for gestational age; SGA, small for gestational age; LGA, large for gestational age; CMR, composite cardiometabolic risk.

a Numbers of cases may not add up to the total value because of missing data.

b Adjusted for age, puberty stage, preterm birth, feeding type, disease during pregnancy, and family history of cardiometabolic disease. The number of patients in the adjusted analyses were different from the unadjusted analyses because factors used for adjustment were not available for all patients.

### 3.3 Comparison of DHEAS levels among CPP girls with different birth size

There was a significant difference in serum DHEAS among SGA, AGA, and LGA patients. After adjustments for age and BMI-SDS, SGA individuals were associated with a 0.21 nmol/L (adjusted β; 95% CI, 0.03 to 0.39) higher mean ln DHEAS than AGA individuals and a 0.28 nmol/L (adjusted β; 95% CI, 0.02 to 0.53) higher mean than LGA individuals. Moreover, high DHEAS level (above the 75th percentile of the whole study group) was found in 36.4% of the SGA individuals, 24.5% of the AGA individuals, and 14.0% of the LGA individuals (*p* = 0.015). The remaining sex hormone concentrations including basic LH, basic FSH, estradiol, peak LH, peak FSH, the ratio of peak LH and FSH, and androstenedione were comparable among the three groups (**Supplementary Figure S2**).

### 3.4 Differences in cardiometabolic risk factors between high and normal DHEAS groups

Next, we wanted to explore the link between DHEAS and cardiometabolic risk factors in CPP girls. Based on the adjusted model, patients in the high DHEAS group had significantly higher HDL cholesterol and apolipoprotein A-1 and lower triglyceride and non-obesity CMR score than their counterparts (**Table 3**). Likewise, in the SGA group, patients with high DHEAS had higher HDL cholesterol (adjusted mean [95% CI], 1.36 [1.26, 1.46] mmol/L vs. 1.23 [1.15, 1.30] mmol/L; *p* = 0.04) and fasting glucose (4.76 [4.65, 4.88] mmol/L vs. 4.59 [4.50, 4.68] mmol/L; *p* = 0.02), and lower ln triglyceride (−0.44 [−0.58, −0.31] mmol/L vs. −0.21 [−0.31, −0.11] mmol/L; *p* = 0.01) and DBP-SDS (0.20 [−0.15, 0.54] vs. 0.80 [0.54, 1.07]; *p* = 0.01). Moreover, in the AGA group, patients with high DHEAS had lower non-obesity CMR score (−0.06 [−0.18, 0.07] vs. 0.13 [0.06, 0.19]; *p* = 0.01).

**Table 3:** Cardiometabolic risk factors in CPP girls according to DHEAS levels.

Variables	Crude			Adjusted		
	Normal DHEAS Mean (95% CI) *n* = 262–321^a^	High DHEAS Mean (95% CI) *n* = 85–110^a^	*p*-Value^†^	Normal DHEAS Mean (95% CI) *n* = 262–321^a^	High DHEAS Mean (95% CI) *n* = 85–110^a^	*p*-Value^‡^
BMI-SDS	0.76 (0.62, 0.90)	1.00 (0.79, 1.20)	0.06	–		
Glucose, mmol/L	4.60 (4.56, 4.65)	4.68 (4.61, 4.76)	0.09	4.62 (4.57, 4.66)	4.66 (4.58, 4.74)	0.40
Insulin^1/2^, mU/L^b^	2.41 (2.33, 2.49)	2.50 (2.39, 2.62)	0.21	2.45 (2.38, 2.52)	2.39 (2.27, 2.51)	0.43
HOMA-IR^1/2 b^	1.09 (1.05, 1.12)	1.15 (1.09, 1.20)	0.11	1.11 (1.08, 1.14)	1.09 (1.04, 1.15)	0.66
HbA1c, %	5.31 (5.28, 5.33)	5.33 (5.29, 5.37)	0.41	5.32 (5.29, 5.34)	5.31 (5.27, 5.36)	0.92
Total cholesterol, mmol/L	3.52 (3.45, 3.59)	3.57 (3.46, 3.68)	0.48	3.51 (3.44, 3.58)	3.59 (3.47, 3.72)	0.26
HDL cholesterol, mmol/L	1.28 (1.26, 1.31)	1.34 (1.29, 1.38)	0.07	1.28 (1.25, 1.30)	1.36 (1.31, 1.41)	0.004
LDL cholesterol, mmol/L	2.11 (2.04, 2.17)	2.10 (2.00, 2.20)	0.84	2.11 (2.05, 2.17)	2.10 (1.99, 2.21)	0.90
Non-HDL-C, mmol/L	2.24 (2.17, 2.30)	2.23 (2.14, 2.33)	0.98	2.24 (2.17, 2.30)	2.24 (2.13, 2.34)	0.99
Ln Triglyceride, mmol/L^c^	−0.29 (−0.34, −0.25)	−0.36 (−0.43, −0.28)	0.15	−0.27 (−0.32, −0.23)	−0.41 (−0.48, −0.34)	0.002
Apolipoprotein A-1, g/L	1.28 (1.26, 1.30)	1.33 (1.29, 1.36)	0.03	1.28 (1.26, 1.30)	1.34 (1.30, 1.38)	0.006
Apolipoprotein B, g/L	0.63 (0.62, 0.65)	0.62 (0.59, 0.64)	0.34	0.64 (0.62, 0.65)	0.62 (0.59, 0.65)	0.39
Ln Lipoprotein (a), nmol/L^c^	2.78 (2.64, 2.93)	2.87 (2.66, 3.08)	0.51	2.79 (2.65, 2.94)	2.84 (2.60, 3.08)	0.74
SBP-SDS	−0.02 (−0.13, 0.08)	0.02 (−0.20, 0.23)	0.71	−0.01 (−0.12, 0.10)	−0.03 (−0.23, 0.16)	0.83
DBP-SDS	0.76 (0.66, 0.86)	0.71 (0.53, 0.88)	0.64	0.76 (0.67, 0.86)	0.69 (0.51, 0.86)	0.47
CMR score	0.21 (0.14, 0.28)	0.22 (0.10, 0.34)	0.93	–		
Non-obesity CMR score	0.10 (0.04, 0.16)	0.05 (−0.07, 0.17)	0.45	0.13 (0.07, 0.19)	−0.05 (−0.15, 0.05)	0.003

A total of 431 patients were included in the analyses. The high DHEAS group was defined as ≥75th percentile value (≥1,290 nmol/L, ≥48 μg/dl) and the normal DHEAS group was defined as <75th percentile value based on overall sample. Statistical significance was based on p < 0.05.

CPP, central precocious puberty; DHEAS, dehydroepiandrosterone sulfate; SDS, standard deviation score; Non-HDL-C, non-high-density lipoprotein cholesterol; SBP, systolic blood pressure; DBP, diastolic blood pressure; CMR, composite cardiometabolic risk.

^†^Independent samples t-test;

^‡^Analysis of covariance, adjusted for age, BW-SDS, and BMI-SDS.

a The number of patients was variable because HbA1c, lipoprotein (a), SBP, DBP, CMR score, and non-obesity CMR score were not available for all patients.

b Variables were square root transformed;

c Variables were natural-log transformed.

### 3.5 Correlation of DHEAS with cardiometabolic risk factors

**Figure 1:**
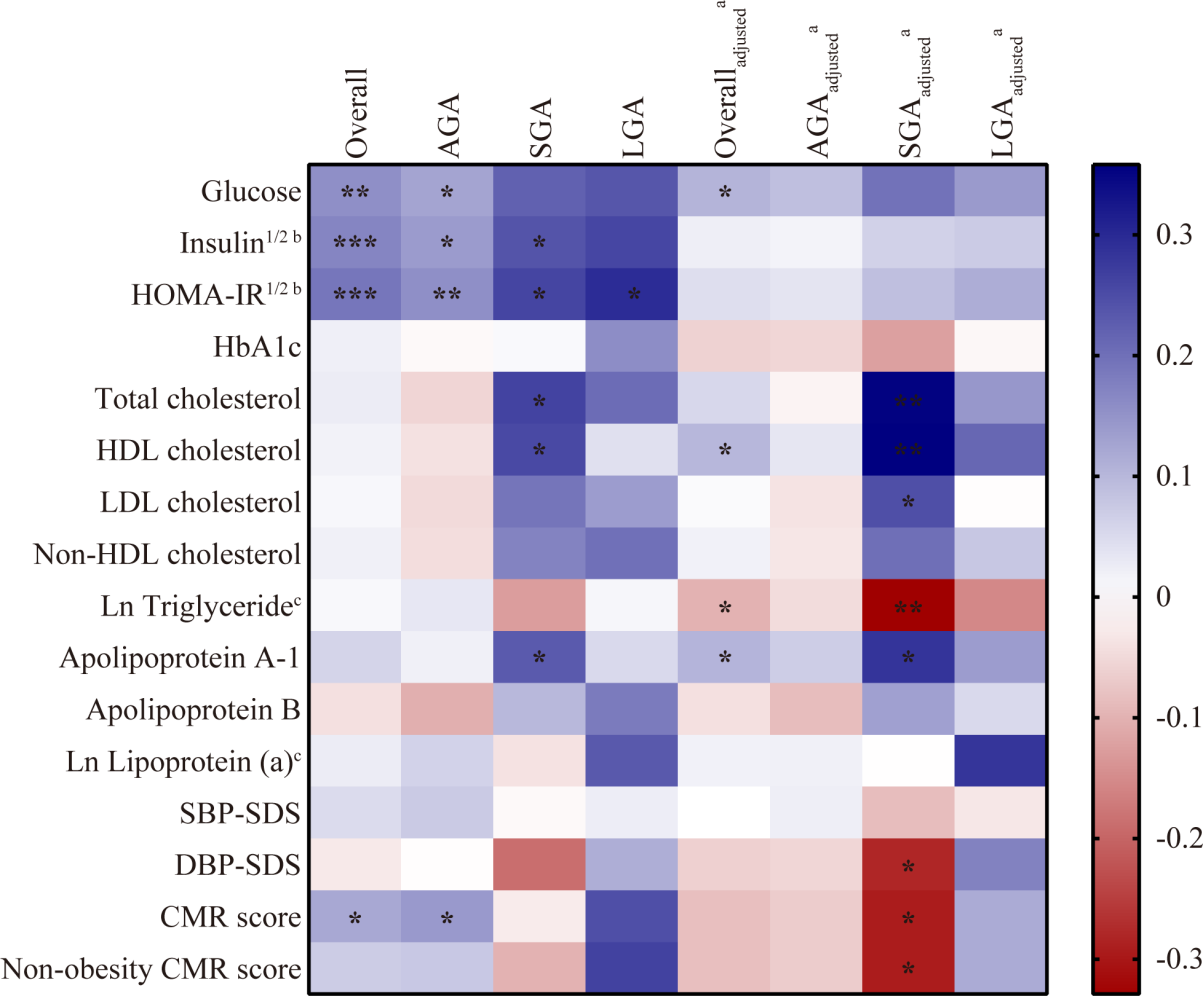
Correlation between DHEAS and cardiometabolic risk factors in CPP girls. Analyzed by partial correlation test. Blue indicated a positive correlation and red indicated a negative correlation with the intensity of the color representing the strength of the correlation. ^*^ *p* < 0.05, ^**^ *p* < 0.01, ^***^ *p* < 0.001. CPP, central precocious puberty; DHEAS, dehydroepiandrosterone sulfate; AGA, appropriate for gestational age; SGA, small for gestational age; LGA, large for gestational age; SBP, systolic blood pressure; DBP, diastolic blood pressure; SDS, standard deviation score; CMR, composite cardiometabolic risk. ^a^adjusted for age, BW-SDS and BMI-SDS. ^b^Variables were square root transformed; ^c^Variables were natural-log transformed.

Partial correlation analysis was performed according to birth weight status. As shown in **Figure 1**, in the overall patients, weak but significant positive correlation was found between DHEAS and fasting glucose (*r* = 0.105, *p* = 0.03), HDL cholesterol (*r* = 0.100, *p* = 0.038), and apolipoprotein A-1 (*r* = 0.104, *p* = 0.032) when controlling for age, BW-SDS, and BMI-SDS. DHEAS negatively correlated with ln triglyceride (*r* = −0.100, *p* = 0.039) after these adjustments. Although DHEAS was positively associated with CMR score (*r* = 0.122, *p* = 0.023) in the unadjusted analysis, the association disappeared when adjusted for confounders. In the SGA group, DHEAS correlated positively with HDL cholesterol (*r* = 0.358, *p* = 0.002), TC (*r* = 0.354, *p* = 0.002), apolipoprotein A-1 (*r* = 0.284, *p* = 0.014), and LDL cholesterol (*r* = 0.247, *p* = 0.034) after multivariate adjustments. Moreover, DHEAS correlated negatively with ln triglyceride (*r* = −0.329, *p* = 0.004), non-obesity CMR score (*r* = –0.293, *p* = 0.033), and DBP-SDS (*r* = −0.280, *p* = 0.042) in the adjusted model. Here, it is important to note that either in the overall participants or in the three categorized groups, DHEAS had a positive association with HOMA-IR, a widely used index of insulin resistance, in the unadjusted model (overall: *r* = 0.191, *p* < 0.001; AGA: *r* = 0.157, *p* = 0.006; SGA: *r* = 0.261, *p* = 0.022; LGA: *r* = 0.295, *p* = 0.038), whereas no significant connection was observed after adjustments for age, BW-SDS, and BMI-SDS. Similarly, before these adjustments, DHEAS positively correlated with insulin in the overall patients (*r* = 0.170, *p* < 0.001), AGA (*r* = 0.140, *p* = 0.015), and SGA group (*r* = 0.240, *p* = 0.036), yet no correlation was significant after correction.

### 3.6 Sensitivity analysis

Propensity score matching (PSM) generated two matched pairs, one including 43 AGA patients and 15 SGA patients, and the other containing 15 AGA patients and 5 LGA patients (**Supplementary Tables S1–S4**, **Figure S3**). All matching variables were balanced between groups. Compared with AGA peers, CPP girls born SGA were associated with a higher likelihood for elevated HbAc1 (3[20.0%] vs. 0[0.0%], *p* = 0.02) and low HDL cholesterol (3[20.0%] vs. 0[0.0%], *p* = 0.02). LGA did not affect the risk of metabolic disorders. The correlation between DHEAS and cardiometabolic risk factors yielded very similar estimates to the original analyses.

## 4 Discussion

Our study showed that CPP girls born SGA were more likely to have elevated HbAc1 and low HDL cholesterol. In contrast, no association between LGA and any glucose or lipid derangements was observed. We found a significant connection between LGA and elevated CMR score, but not on the non-obesity CMR score. Strikingly, after adjustments for age, puberty stage, perinatal information, and family history of cardiometabolic diseases, SGA patients had decreased risk of elevated triglyceride compared to those born AGA. The most interesting finding was that serum DHEAS correlated positively with HDL cholesterol and negatively with triglyceride independent of age, BW-SDS, and BMI-SDS, prominently in SGA individuals.

In line with our result, previous studies found a positive association between SGA and high HbAc1 concentration (31, 32). Moreover, we found that children in the SGA group had 2.41 times the odds to have insulin resistance when compared with the AGA group, although not statistically significant. The sensitive analysis, using stricter definitions and matching current BMI-SDS, also verified the result that glucose metabolism was altered independently of overweight or obesity in SGA children (33).

Evidence regarding lipid outcomes in childhood was inconsistent. Kuhle et al. used data from the Canadian Health Measures Survey to find that birth weight for gestational age did not affect the risk of low HDL cholesterol, which was defined as lower than the 25th percentile (32). However, Sun et al. analyzed data from the National Health and Nutrition Examination Survey to show that low HDL cholesterol, defined as lower than the 5th percentile, was more common among children with low birth weight (31). Differences in ethnic background and cutoff value may contribute to between-study heterogeneities. A relative high cutoff point for abnormal HDL cholesterol may mask the difference at the extreme ranges of the outcome between SGA and AGA status.

The impact of being born LGA on cardiometabolic health was controversial. Chiavaroli et al. indicated that the LGA population had higher CMR score, fasting insulin, and HOMA-IR during childhood that progressed at adolescence (34). However, some studies argued that LGA status presenting with unfavorable metabolic profile was mainly weight-related and being born LGA may protect obesity children against insulin resistance and dyslipidemia (5, 31, 35). To gain better insight into the metabolic characteristics, we calculated CMR score and non-obesity CMR score, respectively. As expected, the difference we observed in the cardiometabolic risk between LGA and AGA status was driven by BMI, suggesting that LGA children may be particularly suitable for targeted interventions to manage weight gain.

Intriguingly, the finding of a negative relationship between SGA status and elevated triglyceride was in disagreement with prior studies (36–38). One possible reason was the influence of body weight. We did not adjust for the variable that could be on the causal pathway to ensure that the main exposure estimates were not biased in the adjusted regression analyses. The comparison between the groups that used PSM partly confirm our hypothesis. On the other hand, the discrepancy may be due to individuals with distinct DHEAS concentration. In agreement with previous research, our study found that serum DHEAS was higher in SGA status than AGA independent of age and BMI-SDS. Furthermore, patients born SGA were more likely to have had pubarche at first visit. In the present study, the differences were accentuated after adjusting potential confounders, implying that DHEAS may have a significant role.

The link between DHEAS and cardiometabolic risk factors still needs further investigation. Prior studies suggested that adrenarche status alone may not be enough to cause adverse metabolic profile at childhood, because metabolic abnormalities mostly occurred in obese subjects (13, 39). Similarly, in our study, a statistically significant positive correlation was observed between DHEAS and HOMA-IR, but the significance was lost after adjustments for age, BW-SDS, and BMI-SDS. Moreover, we found that higher DHEAS was related with beneficial metabolic phenotype among CPP girls. Conversely, Pereira et al. indicated that girls with high DHEAS concentration at age 7 had greater risk for metabolic syndrome at adolescence (1 year after menarche) even after adjustments for adiposity and birth weight (40). On the other hand, the authors found that individuals with high DHEAS had higher androstenedione and free androgen index throughout puberty (11). Notably, a study in PA children found that girls without premature pubarche had milder metabolic changes than their premature pubarche counterparts who showed increased fasting insulin concentration and decreased sex hormone-binding globulin (SHBG) (41). DHEAS as an intermediary of the estradiol and testosterone is a very weak androgen. SHBG is usually used as a measure to judge the severity of hyperandrogenism, because it exhibits high affinity for testosterone. Accumulating lines of evidence have demonstrated a positive connection between serum SHBG and adverse metabolic outcomes (42, 43). Hence, PA children with adverse metabolic outcomes were probably due to increased bioactive androgens rather than DHEAS. Several observations in women with polycystic ovary syndrome provided some evidence that the presence of adrenal hyperandrogenism, defined as high DHEAS concentration, may have a beneficial impact on the lipid profile and blunt the deleterious effect of obesity and high free testosterone (44, 45). Regrettably, we did not measure SHBG concentration in our patients and testosterone concentration was usually undetectable or present at a very low level.

Given that the association between DHEAS and cardiometabolic risk factors may be modified by birth size, previous studies often excluded individuals with low birth weight. Our study showed that among CPP girls born SGA, DHEAS was positively associated with HDL cholesterol and apolipoprotein A-1, and negatively associated with triglyceride, DBP-SDS, and non-obesity CMR score independent of age, birth weight, and current weight. It is well documented that early life growth has an impact on the DHEAS level in childhood (46). Low birth weight followed by early accelerated growth may be adaptive mechanisms to the energy-rich environment and initiated a pattern of events leading to the clustering of metabolic complications, rather than high DHEAS per se. Certainly, there was a possibility that this relation was due to the changes during sexual maturation (14). However, some studies indicated that girls had no significant variation in HDL cholesterol and triglyceride levels through pubertal stage (47, 48). As for the positive relationship between DHEAS and LDL cholesterol in SGA status, preterm birth and low SHBG level may be one of the confounders. Ibáñez et al. showed that increased DHEAS was the main variable associated with decreased SHBG among children born SGA (49).

These findings may have clinical implications. Cardiometabolic risk factors can be tracked from childhood to adulthood. Among CPP girls, individuals born SGA were more likely to possess cardiometabolic risk factors compared to their counterparts born AGA. Early preventive evaluation should therefore be considered in this high-risk group. Moreover, taken together with our prior study, children born SGA have grown in importance in evaluation for CPP (30). It reminds the clinicians to obtain comprehensive medical history to identify these children and provide more attention and support. On the other hand, there is a positive correlation between adiposity and lipid derangements in childhood and adulthood. We observed that the difference in cardiometabolic risk between individuals born LGA and AGA was driven by BMI. Furthermore, individuals with obesity had almost triple the risk of having an elevated non-obesity CMR score than those with normal weight in CPP girls born LGA (6 [40.0%] vs. 3 [18.8%], OR = 2.89). Hence, CPP girls with LGA status may need early nutritional and lifestyle intervention aimed at preventing weight-related complications. Additionally, the results may provide some evidence that the protective effect of DHEAS on cardiometabolic condition can be found in CPP girls born SGA. Among adults, consolidated data show that the DHEA and DHEAS present protective actions on the cardiovascular system and could be used as a drug for cardiovascular disease (50). Future studies should evaluate whether this protective effect is persistent throughout childhood and adolescence.

This study has several strengths. We included multiple markers of cardiometabolic outcomes and calculated a continuous CMR score that may better capture potential disease risk. Moreover, complications during pregnancy, positive family history of cardiometabolic disease, preterm birth, and feeding practices in infancy may be associated with increased cardiometabolic risk later in life. We controlled for these potential confounding variables. Furthermore, we investigated the relationship between DHEAS and cardiometabolic risk factors in CPP girls born SGA and LGA.

The study also has some limitations. There might have been recall bias pertaining to parental recollection of birth weight and gestational age. However, a previous study indicated that misclassification could have little influence on the results (51). In addition, DHEAS was measured with an immunoassay not with liquid chromatography–tandem mass spectrometry. However, it barely affected the results, because DHEAS concentrations in our sample were usually sufficiently high to be detected by immunoassays. As a result of being a retrospective investigation, some information was not recorded or missing, which may contribute to the lack of association found or overstate our findings. The potential impact of missing data was assessed in sensitivity analyses. Moreover, the correlation coefficients between DHEAS and cardiometabolic risk markers (although in line with other cohorts in the literature) were relatively small (14, 52). A prospective study with large samples is required in the future. Lastly, there may have been interobserver variability in assessing anthropometric and blood pressure measurements, but these examinations were normally performed by well-trained professionals.

In summary, among CPP girls, compared with AGA counterparts, individuals born SGA were associated with higher risk of abnormal glucose metabolism and dyslipidemia at the time of diagnosis. The difference in cardiometabolic risk between LGA and AGA status was driven by BMI. It has a beneficial impact of DHEAS on the lipid metabolism in CPP girls even in those who were born SGA, evidenced by an association with higher HDL cholesterol and lower triglyceride.

## Data availability statement

The raw data supporting the conclusions of this article will be made available by the corresponding author, without undue reservation.

## Ethics statement

The studies involving human participants were reviewed and approved by the Ethics Committee of Tongji Hospital of Tongji Medical College of Huazhong University of Science and Technology (TJ-IRB20211011). Written informed consent to participate in this study was provided by the participants’ legal guardian/next of kin.

## Author contributions

GZ analyzed the data and drafted the manuscript. HY and SY took part in the collection of clinical data. XL, YL, and LH had substantial contributions to the acquisition of data. WW contributed to the concept and design of the study, and revised the article critically for relevant knowledgeable content. All authors contributed to the article and approved the submitted version.
