# Supplementary material for: Association of size for gestational age and dehydroepiandrosterone sulfate with cardiometabolic risk in central precocious puberty girls
Source: Front Endocrinol (Lausanne). 2023 May 24;14:1131438. doi: 10.3389/fendo.2023.1131438 (PMC10244634; doi:10.3389/fendo.2023.1131438)
Supplement: Supplementary file 1 [file Image_1.pdf]

Figure S1. Flowchart of Participant Enrollment.

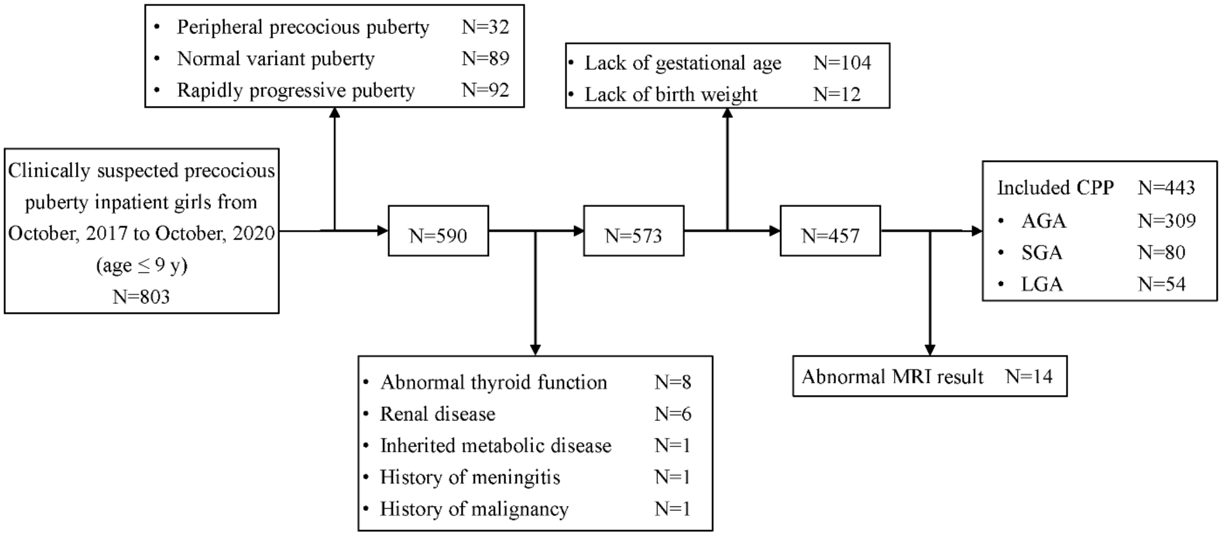

Abbreviations: CPP, central precocious puberty; AGA, appropriate for gestational age; SGA, small for gestational age; LGA, large for gestational age; MRI, magnetic resonance imaging.
