# Supplementary material for: Association of size for gestational age and dehydroepiandrosterone sulfate with cardiometabolic risk in central precocious puberty girls
Source: Front Endocrinol (Lausanne). 2023 May 24;14:1131438. doi: 10.3389/fendo.2023.1131438 (PMC10244634; doi:10.3389/fendo.2023.1131438)
Supplement: Supplementary file 2 [file Image_2.pdf]

Figure S2. Sex Hormonal Profile by Size for Gestational Age in CPP Girls.

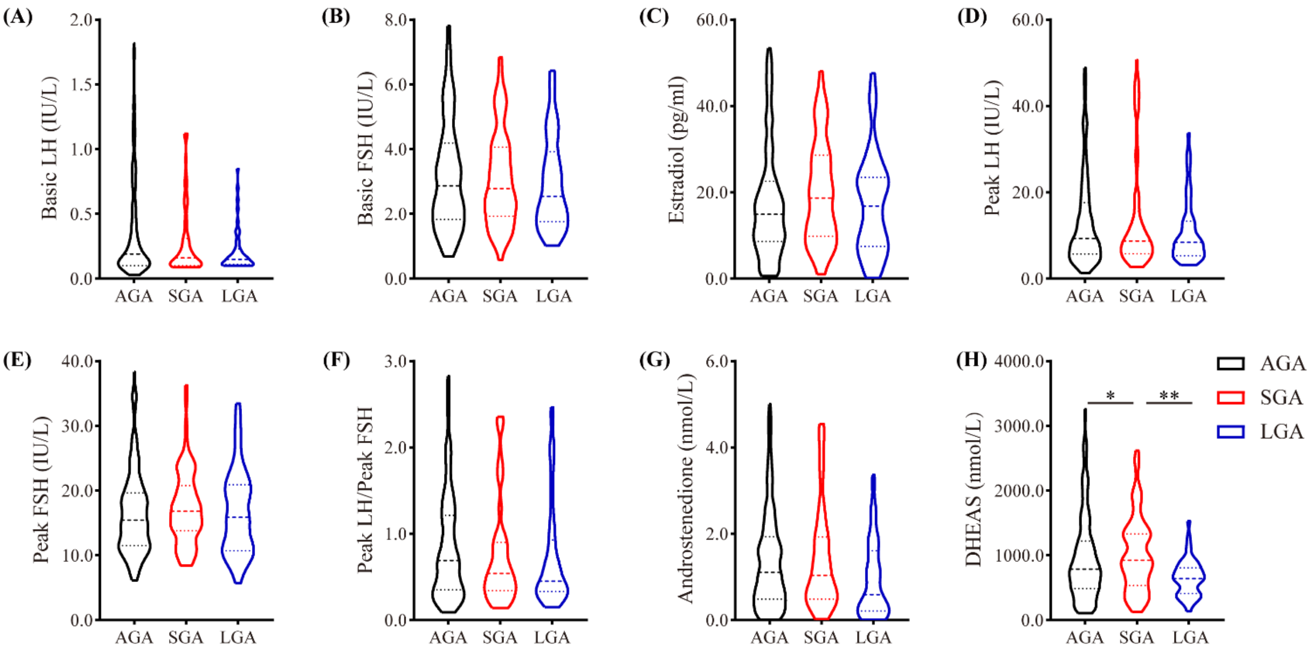

Analyzed by Kruskal-Wallis nonparametric test. $^{*}P$ <0.05, $^{**}P$ <0.01. The number of patients varied from 431 to 443 because some DHEAS data were missing.

Abbreviations: CPP, central precocious puberty; AGA, appropriate for gestational age; SGA, small for gestational age; LGA, large for gestational age; LH, luteinizing hormone; FSH, follicle stimulating hormone; DHEAS, dehydroepiandrosterone sulfate.
