# Supplementary material for: Association of size for gestational age and dehydroepiandrosterone sulfate with cardiometabolic risk in central precocious puberty girls
Source: Front Endocrinol (Lausanne). 2023 May 24;14:1131438. doi: 10.3389/fendo.2023.1131438 (PMC10244634; doi:10.3389/fendo.2023.1131438)
Supplement: Supplementary file 3 [file Image_3.pdf]

Figure S3. Correlation between DHEAS and Cardiometabolic Risk Factors in CPP Girls after PSM.

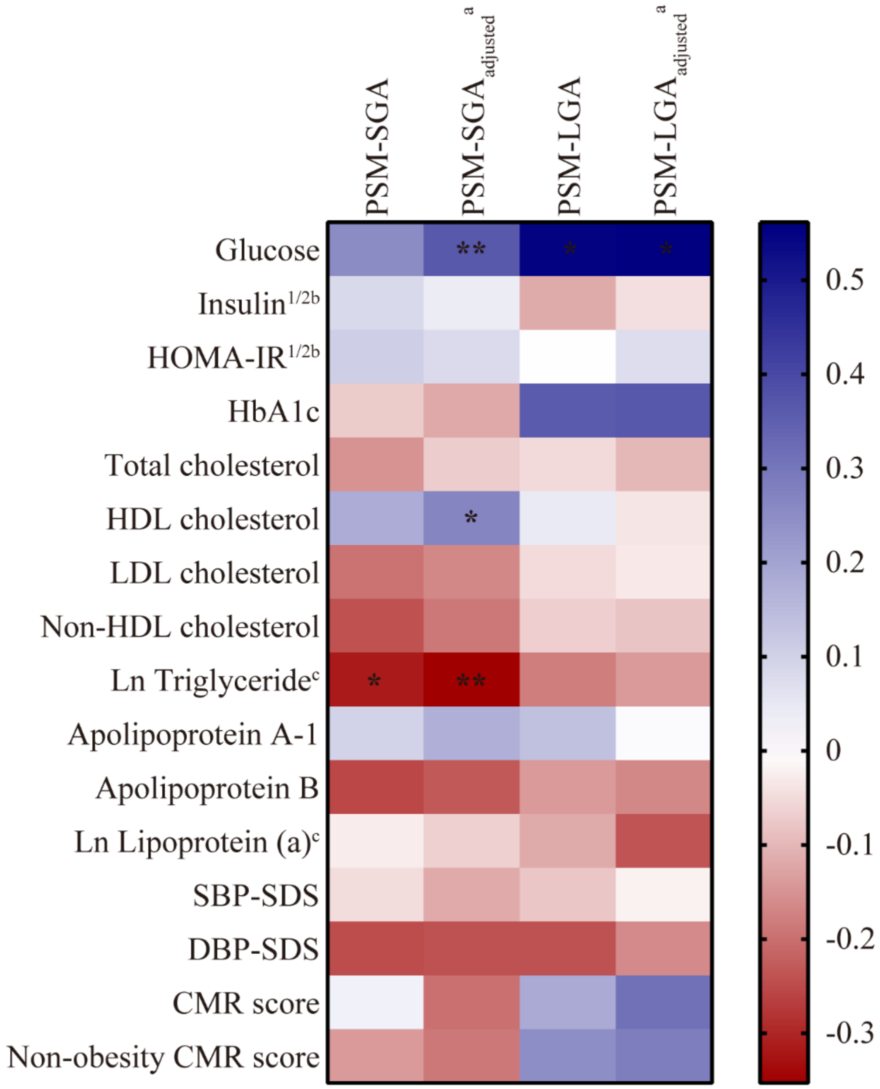

Analyzed by partial correlation test. Blue indicated a positive correlation and red indicated a negative correlation with the intensity of the color representing the strength of the correlation. $^{*}P$ <0.05, $^{**}P$ <0.01.

Abbreviations: CPP, central precocious puberty; AGA, appropriate for gestational age; SGA, small for gestational age; LGA, large for gestational age; PSM-SGA, AGA and SGA individuals after propensity score matching; PSM-LGA, AGA and LGA individuals after propensity score matching; SBP, systolic blood pressure; DBP, diastolic blood pressure; SDS, standard deviation score; CMR, composite cardiometabolic risk.

[a]adjusted for age, BW-SDS and BMI-SDS. [b]Variables were square root transformed; [c]Variables were natural-log transformed.
