# Supplementary material for: Association of size for gestational age and dehydroepiandrosterone sulfate with cardiometabolic risk in central precocious puberty girls
Source: Front Endocrinol (Lausanne). 2023 May 24;14:1131438. doi: 10.3389/fendo.2023.1131438 (PMC10244634; doi:10.3389/fendo.2023.1131438)
Supplement: Supplementary file 4 [file Table_1.docx]

**Table S1.** General Characteristics between CPP Girls Born AGA and SGA after PSM.

| **Characteristics** | **AGA (n=43)** | **SGA (n=15)** | ***P* Value** |
| --- | --- | --- | --- |
| **Child** |  |  |  |
| age, Median (IQR), y | 7.5 (7.0, 8.0) | 7.4 (7.0, 8.0) | 0.86^†^ |
| Height-for-age, Mean ± SD, cm | 128.2 ± 6.2 | 122.6 ± 5.9 | 0.003^‡^ |
| Height-for-age SDS, Mean ± SD | 0.63 ± 1.39 | -0.45 ± 0.78 | 0.006^‡^ |
| THt-SDS, Mean ± SD | -0.28 ± 0.74 | -0.67 ± 0.73 | 0.08^‡^ |
| PAH-SDS _THt-SDS_, Mean ± SD | -0.99 ± 1.11 | -1.67 ± 0.88 | 0.04^‡^ |
| Weight, Median (IQR), kg | 24.5 (22.0, 26.5) | 23.0 (20.0, 24.5) | 0.06^†^ |
| BMI, Mean ± SD, kg/m^2^ | 15.1 ± 1.6 | 15.2 ± 1.5 | 0.88^‡^ |
| BMI-SDS, Mean ± SD | -0.12 ± 1.12 | -0.02 ± 0.96 | 0.76^‡^ |
| BMI status, n (%) |  |  | 0.34^§^ |
| Not overweight or obesity | 39 (90.7%) | 13 (86.7%) |  |
| Overweight | 0 (0.0%) | 1 (6.7%) |  |
| Obesity | 4 (9.3%) | 1 (6.7%) |  |
| Breast Tanner stage 2, n (%) | 43 (100.0%) | 15 (100.0%) | - |
| Pubarche, n (%) | 0 (0.0%) | 0 (0.0%) | - |
| Menarche, n (%) | 0 (0.0%) | 0 (0.0%) | - |
| **Perinatal** |  |  |  |
| Gestational age, Median (IQR), wk | 39.0 (38.7, 40.0) | 38.0 (37.7, 40.0) | 0.77^†^ |
| Birth weight, Mean ± SD, kg | 3.2 ± 0.27 | 2.3 ± 0.18 | <0.001^‡^ |
| Birth weight SDS, Mean ± SD | -0.14 ± 0.61 | -2.39 ± 0.20 | <0.001^‡^ |
| GDM or GH, n (%) | 0 (0.0%) | 0 (0.0%) | - |
| Primiparous, n (%) | 37 (86.0%) | 11 (73.3%) | 0.27^§^ |
| Caesarean delivery, n (%) | 28 (65.1%) | 10 (66.7%) | 0.59^¶^ |
| Infant feeding, n (%) |  |  | 0.84^§^ |
| Exclusive breasting | 24 (55.8%) | 7 (46.7%) |  |
| Formula feeding | 14 (32.6%) | 6 (40.0%) |  |
| Mixed feeding | 5 (11.6%) | 2 (13.3%) |  |
| **Family history** |  |  |  |
| Cardiometabolic risk, n (%) |  |  | 0.59^§^ |
| Parents | 0 (0%) | 0 (0.0%) |  |
| Grandparents | 8 (18.6%) | 3 (20.0%) |  |
| No | 35 (81.4%) | 12 (80.0%) |  |

Analyzed by propensity score matching (PSM). Matching items of PSM consisted of child’s age, BMI-SDS, puberty stage, feeding pattern, disease during pregnancy and family history of cardiometabolic disease. Statistically significance was based upon *P* value less than 0.05.

Abbreviations: CPP, central precocious puberty; AGA, appropriate for gestational age; SGA, small for gestational age; PSM, propensity score matching; SDS, standard deviation score; THt, genetic target height; PAH, Predictive adult height; PAH-SDS _THt-SDS_, the loss in height potential; BMI, body mass index; GDM, gestational diabetes mellitus; GH, gestational hypertension.

^†^Mann-Whitney U test; ^‡^Independent samples t-test; ^§^Fisher exact test; ^¶^Chi-square test.
