# Supplementary material for: Association of size for gestational age and dehydroepiandrosterone sulfate with cardiometabolic risk in central precocious puberty girls
Source: Front Endocrinol (Lausanne). 2023 May 24;14:1131438. doi: 10.3389/fendo.2023.1131438 (PMC10244634; doi:10.3389/fendo.2023.1131438)
Supplement: Supplementary file 6 [file Table_3.docx]

**Table S3.** General Characteristics between CPP girls Born AGA and LGA after PSM.

| **Characteristics** | **AGA (n=15)** | **LGA (n=5)** | ***P* Value** |
| --- | --- | --- | --- |
| **Child** |  |  |  |
| age, Mean ± SD, y | 8.00 ± 0.81 | 8.03 ± 0.66 | 0.94^†^ |
| Height-for-age, Mean ± SD, cm | 131.2 ± 7.0 | 132.9 ± 7.3 | 0.66^†^ |
| Height-for-age-SDS, Mean ± SD | 0.53 ± 1.08 | 0.83 ± 1.13 | 0.60^†^ |
| THt-SDS, Mean ± SD | -0.43 ± 0.74 | -0.26 ± 0.62 | 0.66^†^ |
| PAH-SDS _THt-SDS_, Mean ± SD | -0.96 ± 0.87 | -1.11 ± 1.00 | 0.74^†^ |
| Weight, Mean ± SD, kg | 29.7 ± 4.5 | 30.3 ± 5.0 | 0.80^†^ |
| BMI, Mean ± SD, kg/m^2^ | 17.2 ± 1.4 | 17.1 ± 2.1 | 0.95^†^ |
| BMI-SDS, Mean ± SD | 1.09 ± 0.78 | 1.07 ± 1.32 | 0.98^†^ |
| BMI status, n (%) |  |  | 0.82^§^ |
| Not overweight or obesity | 7 (46.7%) | 3 (60.0%) |  |
| Overweight | 3 (20.0%) | 0 (0.0%) |  |
| Obesity | 5 (33.3%) | 2 (40.0%) |  |
| Breast Tanner stage, n (%) |  |  | 1.00^§^ |
| 2 | 12 (80.0%) | 4 (80.0%) |  |
| 3 | 3 (20.0%) | 1 (20.0%) |  |
| 4 | 0 (0.0%) | 0 (0.0%) |  |
| Pubarche, n (%) | 0 (0.0%) | 0 (0.0%) | - |
| Menarche, n (%) | 0 (0.0%) | 0 (0.0%) | - |
| **Perinatal** |  |  |  |
| Gestational age, Median (IQR), w | 40.0 (38.4, 40.6) | 40.0 (39.5, 40.2) | 0.87^‡^ |
| Birth weight, Mean ± SD, kg | 3.1 ± 0.23 | 4.2 ± 0.10 | <0.001^†^ |
| Birth weight SDS, Mean ± SD | -0.27 ± 0.46 | 2.52 ± 0.29 | <0.001^†^ |
| Infant feeding, n (%) |  |  | 1.00^§^ |
| Exclusive breasting | 12 (80.0%) | 4 (80.0%) |  |
| Formula feeding | 0 (0.0%) | 0 (0.0%) |  |
| Mixed feeding | 3 (20.0%) | 1 (20.0%) |  |
| GDM or GH, n (%) | 0 (0.0%) | 0 (0.0%) | - |
| Primiparous, n (%) | 10 (66.7%) | 4 (80.0%) | 1.00^§^ |
| Caesarean delivery, n (%) | 7 (46.7%) | 5 (100.0%) | 0.06^§^ |
| **Family history** |  |  |  |
| Cardiometabolic risk, n (%) |  |  | 1.00^§^ |
| Parents | 0 (0%) | 0 (0.0%) |  |
| Grandparents | 2 (13.3%) | 1 (20.0%) |  |
| No | 13 (86.7%) | 4 (80.0%) |  |

Analyzed by propensity score matching (PSM). Matching items of PSM consisted of child’s age, BMI-SDS, puberty stage, feeding pattern, disease during pregnancy and family history of cardiometabolic disease. Statistically significance was based upon *P* value less than 0.05.

Abbreviations: CPP, central precocious puberty; AGA, appropriate for gestational age; LGA, large for gestational age; PSM, propensity score matching; SDS, standard deviation score; THt, genetic target height; PAH, Predictive adult height; PAH-SDS _THt-SDS_, the loss in height potential; BMI, body mass index; GDM, gestational diabetes mellitus; GH, gestational hypertension.

^†^Independent samples t-test; ^‡^Mann-Whitney U test; ^§^Fisher exact test
