# Supplementary material for: Association of size for gestational age and dehydroepiandrosterone sulfate with cardiometabolic risk in central precocious puberty girls
Source: Front Endocrinol (Lausanne). 2023 May 24;14:1131438. doi: 10.3389/fendo.2023.1131438 (PMC10244634; doi:10.3389/fendo.2023.1131438)
Supplement: Supplementary file 7 [file Table_4.docx]

**Table S4.** Adverse Levels of Cardiometabolic Risk Factors between CPP girls Born AGA and LGA after PSM.

| **Variable** | **AGA (n=15)** | **LGA (n=5)** | ***P* value** |
| --- | --- | --- | --- |
| BMI ≥95th | 5 (33.3%) | 2 (40.0%) | 1.00 |
| Glucose ≥5.60 mmol/L | 0 (0.0%) | 1 (20.0%) | 0.25 |
| HOMA-IR >3 | 0 (0.0%) | 1 (20.0%) | 0.25 |
| HDL cholesterol <1.04 mmol/L | 1 (6.7%) | 0 (0.0%) | 1.00 |
| LDL cholesterol ≥3.37 mmol/L | 1 (6.7%) | 0 (0.0%) | 1.00 |
| Non-HDL cholesterol ≥3.76 mmol/L | 1 (6.7%) | 0 (0.0%) | 1.00 |
| Triglyceride ≥1.13 mmol/L | 1 (6.7%) | 1 (20.0%) | 0.45 |
| Apolipoprotein A-1 <1.15 g/L | 0 (0.0%) | 1 (20.0%) | 0.25 |
| Lipoprotein (a) ≥72 nmol/L | 3 (20.0%) | 0 (0.0%) | 0.54 |
| Blood Pressure ≥95th | 1 (6.7%) | 0 (0.0%) | 1.00 |
| Elevated CMR score | 2 (13.3%) | 2 (40.0%) | 0.25 |
| Elevated non-obesity CMR score | 1 (6.7%) | 1 (20.0%) | 0.45 |

Analyzed by Fisher exact test. Statistically significance was based upon *P* <0.05. No AGA or LGA patients met the criteria for HbA1c >5.7%, total cholesterol ≥5.18 mmol/L or apolipoprotein B ≥1.1g/L.

Abbreviations: CPP, central precocious puberty; AGA, appropriate for gestational age; LGA, large for gestational age; PSM, propensity score matching; CMR, composite cardiometabolic risk.
